# Supplementary material for: COVID-19 spread, detection, and dynamics in Bogota, Colombia
Source: Nat Commun. 2021 Aug 5;12:4726. doi: 10.1038/s41467-021-25038-z (PMC8342514; doi:10.1038/s41467-021-25038-z)
Supplement: Supplementary file 1 — Supplementary Information [file 41467_2021_25038_MOESM1_ESM.pdf]

Supplementary Materials to "COVID-19 spread, detection, and dynamics in Bogotá, Colombia"

Rachid Laajaj*[1], Camilo De Los Rios[2], Ignacio Sarmiento-Barbieri[1], Danilo Aristizabal[1], Eduardo Behrentz[1], Raquel Bernal[1], Giancarlo Buitrago[3,4], Zulma Cucunubá[5,6], Fernando de la Hoz[3], Alejandro Gaviria[1], Luis Jorge Hernández[1], Leonardo León[1], Diane Moyano[7], Elkin Osorio[7], Andrea Ramírez Varela[1], Silvia Restrepo[1], Rodrigo Rodriguez[7], Norbert Schady[8], Martha Vives[1], and Duncan Webb[9]

[1]University of Los Andes, Bogotá, Colombia
[2]Inter-American Development Bank, Washington D.C, United States
[3]Universidad Nacional de Colombia, Bogotá, Colombia
[4]Hospital Universitario Nacional de Colombia, Bogotá, Colombia
[5]Imperial College London, London, UK
[6]Universidad Pontificia Javeriana, Bogotá, Colombia
[7]Secretaria de Salud de Bogotá, Bogotá, Colombia
[8]World Bank, Washington D.C, United States
[9]Paris School of Economics, Paris, France

*r.laajaj@uniandes.edu.co

Supplementary Note 1

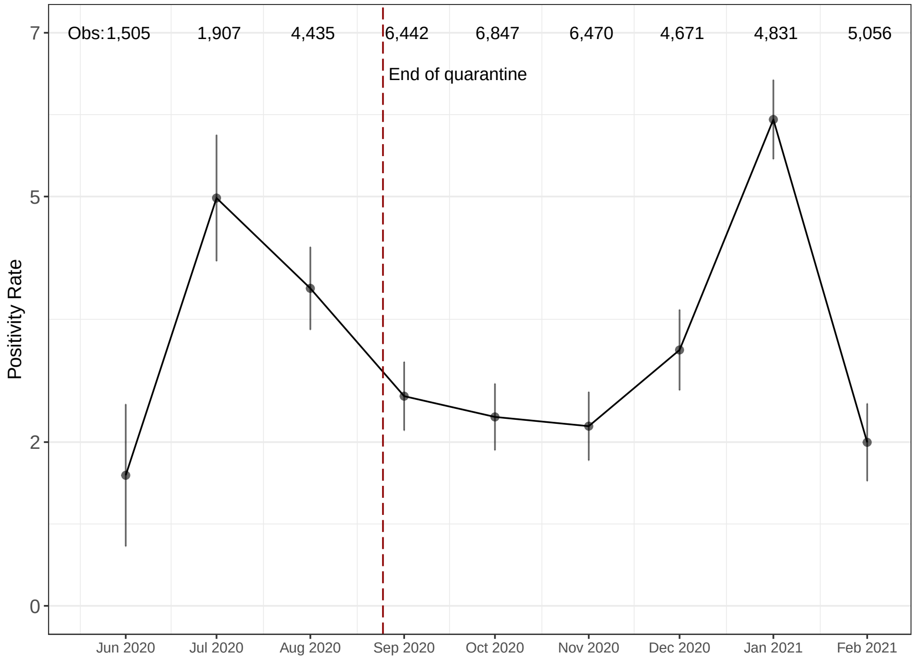

Supplementary Figure 1. **Raw Positivity.** The figure shows the unweighted raw positivity rate by month. The number of observations is provided at the top of the figure. Whiskers denote 95% confidence intervals.

Supplementary Table 1. Positivity rate using different CoVIDA subsamples

Sample	% Positivity Weighted	% Positivity Non-Weighted	Observations
Full sample	5.75<br>(5.57 - 5.94)	5.08<br>(4.91 - 5.26)	59,770
Excluding symptomatic and/or known contact*	3.08<br>(2.92 - 3.25)	3.06<br>(2.90 - 3.22)	42,164
Only symptomatic and/or known contact	11.97<br>(11.49 - 12.45)	9.93<br>(9.49 - 10.38)	17,606
Participants that we invited (from lists)	3.24<br>(3.00 - 3.49)	3.20<br>(2.96 - 3.44)	20,496
Participants from public campaign	2.99<br>(2.76 - 3.21)	2.93<br>(2.70 - 3.15)	21,668

Note: Estimated positivity using CoVIDA data were calculated using a an aggregated average and assuming a 17 day positivity window. Weights were calculated based on workers' occupation. Population of workers category was obtained from a review of official records from several sources. Analytical 95% Confidence Intervals in parentheses. Given an outbreak on a military battalion (69 positives out of 135) , all tested on the 2nd of July, all the samples exclude this battalion. * This is the main sample used for the estimations in the paper.

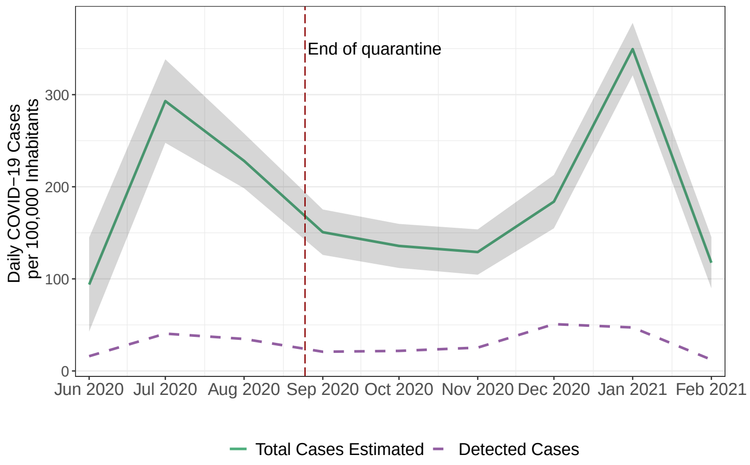

(a) Daily Cases per 100,000 Inhabitants

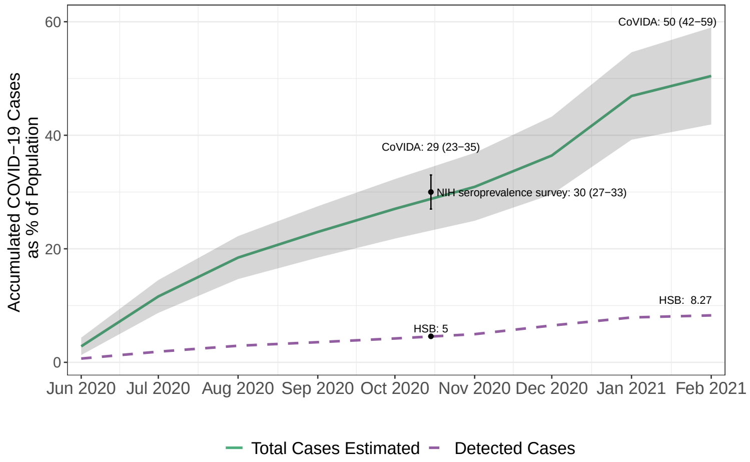

(b) Accumulated Cases as % of Population

Supplementary Figure 2. **Unweighted estimates of daily new cases of COVID-19 per 100,000 inhabitant and accumulated cases as % of Population.** Replicates Figure 1 unweighted. i.e. omits the use of occupations weights. (a) Total daily COVID-19 cases per 100,000 inhabitants based on CoVIDA data and detected cases based on data from the Health Secretary of Bogotá (HSB). The vertical dashed line marks the end of quarantine on August 25, 2020. (b) Cumulative cases as % of Bogotá's population. In black the point estimate and 95% confidence interval of the test positivity rate from a seroprevalence survey run by the National Health Institute of Colombia (NHI) (1). Green solid line in panel (a) represents estimates of the number of daily cases per 100,000 inhabitants for each month (n=42,164). The purple dashed line in panel (a) represents the number of cases per 100,000 inhabitants reported by the HSB. Green solid line in panel (b) represents the accumulated estimates of daily number of cases in panel (a) % of population. Shaded area denotes 95% confidence intervals. Dashed purple line represents the accumulated number of cases as % of population reported by HSB.

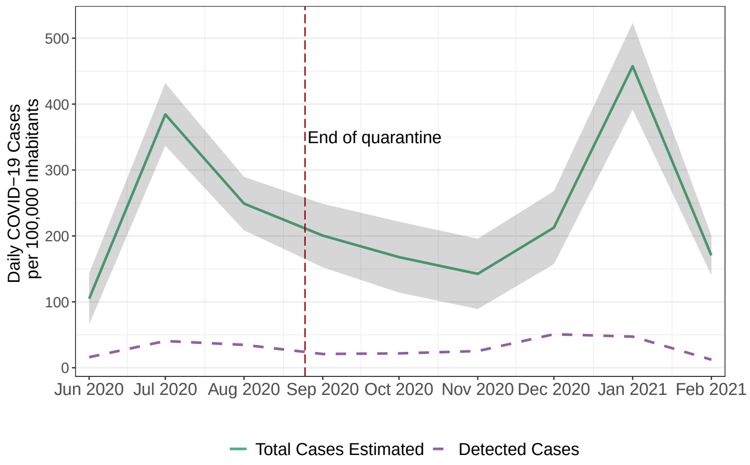

(a) Daily Cases per 100,000 Inhabitants

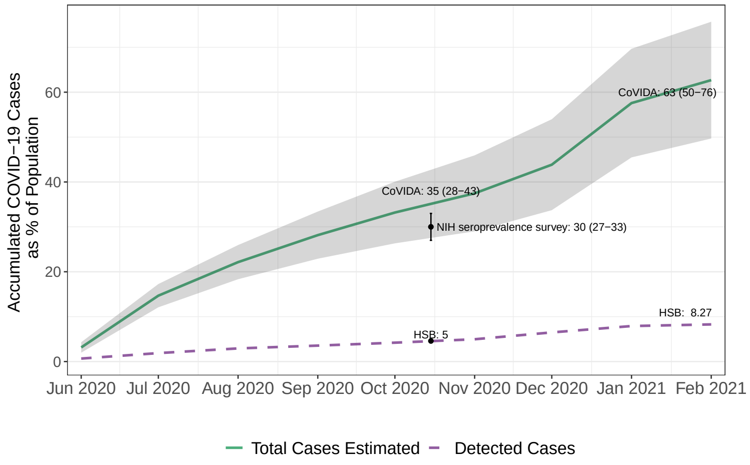

(b) Accumulated Cases as % of Population

Supplementary Figure 3. **Sample restricted daily new cases of COVID-19 per 100,000 inhabitant and accumulated cases as % of Population.** Replicates Figure 1 but excludes participants from the public campaign. (a) Total daily COVID-19 cases per 100,000 inhabitants based on CoVIDA data and detected cases based on data from the Health Secretary of Bogotá (HSB). The vertical dashed line marks the end of quarantine on August 25, 2020. (b) Cumulative cases as % of Bogotá's population. In black the point estimate and 95% confidence interval of the test positivity rate from a seroprevalence survey run by the National Health Institute of Colombia (NHI) (1). Green solid line in panel (a) represents the weighted estimates of the number of daily cases per 100,000 inhabitants for each month (n=20,496). The purple dashed line in panel (a) represents the number of cases per 100,000 inhabitants reported by the HSB. Green solid line in panel (b) represents the weighted accumulated estimated daily number of cases in panel (a) % of population. Weights are based on workers' occupation to be representative of Bogotá's population. Shaded area denotes 95% confidence intervals. Dashed purple line represents the accumulated number of cases as % of population reported by HSB.

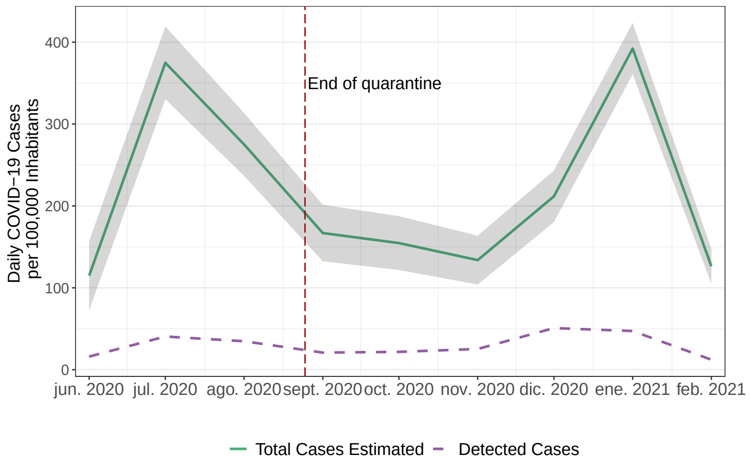

(a) Daily Cases per 100,000 Inhabitants

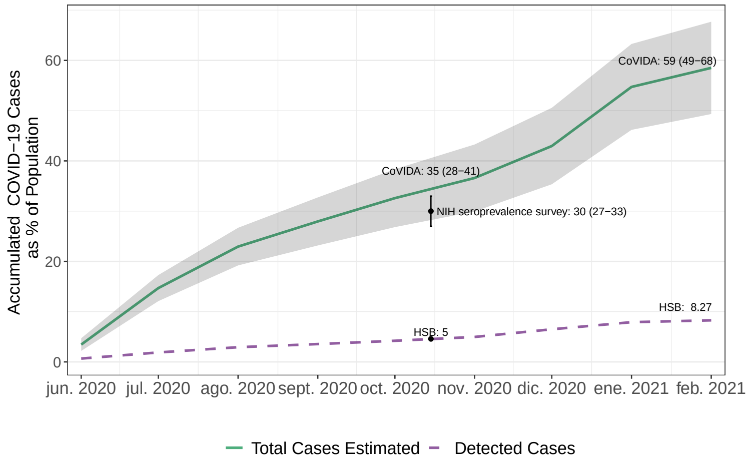

(b) Accumulated Cases as % of Population

Supplementary Figure 4. **Robustness to number of days that an individual can test positive.** Replicates Figure 1 assuming that a person can test positive for 15.5 days. (a) Total daily COVID-19 cases per 100,000 inhabitants based on CoVIDA data and detected cases based on data from the Health Secretary of Bogotá (HSB). The vertical dashed line marks the end of quarantine on August 25, 2020. (b) Cumulative cases as % of Bogotá's population. In black the point estimate and 95% confidence interval of the test positivity rate from a seroprevalence survey run by the National Health Institute of Colombia (NHI) (1). Green solid line in panel (a) represents the weighted estimates of the number of daily cases per 100,000 inhabitants for each month (n=42,164). The purple dashed line in panel (a) represents the number of cases per 100,000 inhabitants reported by the HSB. Green solid line in panel (b) represents the weighted accumulated estimated daily number of cases in panel (a) % of population. Weights are based on workers' occupation to be representative of Bogotá's population. Shaded area denotes 95% confidence intervals. Dashed purple line represents the accumulated number of cases as % of population reported by HSB.

Supplementary Note 2

Here we calculate the value of R_q, which we define as the average number of secondary infections generated by an infected individual at the start of the generalized quarantine period under the assumption that the proportion of susceptible individuals is 1. We treat this value as a constant in the early phase of the quarantine. To calculate the value of R_q, we use the Lotka-Euler equation (2). This assumes exponential growth in new cases, assumes that all individuals in the population are susceptible ($S = 1$), and uses the rate of exponential growth in new cases r and the distribution of the generation interval $g(a)$ to calculate an estimate of R_q.

First, we calculate the rate of exponential growth in new confirmed cases per day by running an OLS regression with the natural log of daily confirmed cases as the outcome variable, and the date as the independent variable. We limit our sample to the early period of the epidemic April 1st 2020 to June 1st 2020, when the exponential growth curve fits the data well and when immunity is unlikely to play a role in case growth because $S \approx 1$. This yields an estimate of r = 0.038 (95% CI: 0.035, 0.041). Figure 5 displays the log daily confirmed new cases in Bogotá over time (the gray dots), and plots the line of best fit (in red) whose slope is equal to r.

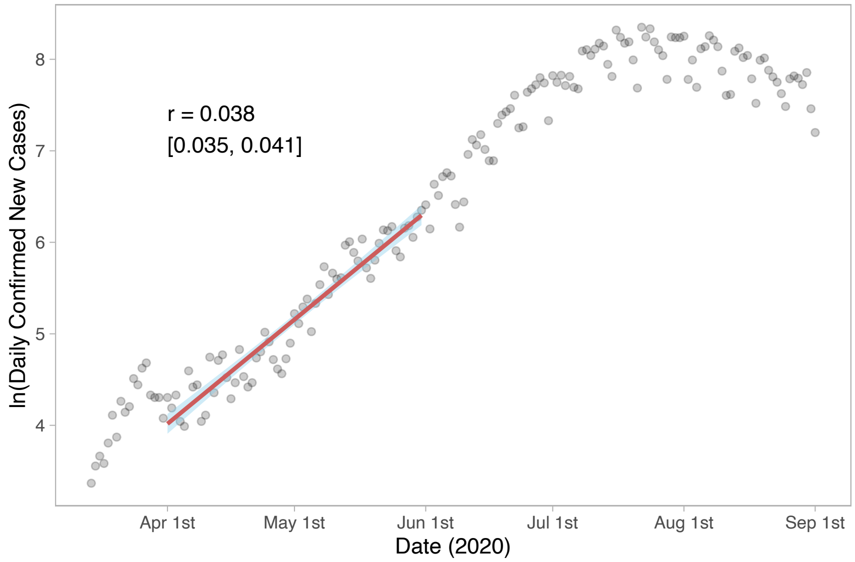

Supplementary Figure 5. **Estimate of the exponential rate of growth in new cases r.** Dots represent log daily confirmed new cases in Bogotá between March 14th, 2020 and September 1st, 2020 (n=172). Red line represents the fitted OLS regression between April 1st, 2020 and May 31st, 2020. Blue shaded areas represent the 95% confidence interval.

Second, we assume that the generation interval $g(a)$, where a is the number of days since infection, is described by a gamma distribution with a mean of 5.2 days and a shape parameter of 4.79. The choice of these parameters reflects an estimation process seen in a companion paper (3), in which we calibrate the generation interval based on data for the serial interval (4)

and the incubation period (5). Moreover, our mean of 5.2 days falls within the confidence range seen in a recent meta-analysis (6) , which estimated the mean generation interval to be 4.8 [95% CI 4.3-5.41] when using a fitted gamma distribution.

Using the estimated value of r, and the assumed distribution for $g(a)$, we calculate the initial value of R_q using the Lotka-Euler equation:

$$\frac{1}{R_q} = \int_{a=0}^{\infty} e^{(-ra)} g(a) da \tag{1}$$

Where $g(a)$ is the density of the generation interval as a function of the day since infection a, r is the rate of exponential growth in new cases.

Using the values of $r = 0.038$ and the assumed $g(a)$ function, this yields an estimate of R_q = 1.218 [95% CI: 1.172, 1.266]. Note that this estimate comes during a period of strict lockdown in Bogotá, which explains why our estimate of R_q is significantly lower than the estimates of R_0 seen in the literature (which are typically calculated in conditions of full mobility (7)).

Supplementary Note 3

Supplementary Table 2. Number of Tests by Month and Invitation Type

Month	Invited Lists	Public Campaign	Total
June 2020	1,505	0	1,505
July 2020	1,490	417	1,907
August 2020	3,273	1,162	4,435
September 2020	3,175	3,267	6,442
October 2020	2,679	4,168	6,847
November 2020	2,284	4,186	6,470
December 2020	1,887	2,784	4,671
January 2021	1,467	3,364	4,831
February 2021	2,736	2,320	5,056
Total	20,496	21,668	42,164

Note: Table shows number of tests by month and invitation type. February include the first 3 days in March. Invited lists contain participants obtained through various agreements with partners. Many of the lists represented a large share of a given occupation, with the aim of obtaining a representative sample within each occupation. Public campaign contains participants from a widely disseminated public invitation for free testing. The sample excludes individuals with symptoms or known contacts with an infected person.

Supplementary Table 3. One case detected out of...

Month	Stratum				Average
	1&2	3	4	5&6	
June	2.4	8.3	1.2	7.6	6.5
	(0.0 - 7.7)	(0.0 - 17.6)	(0.0 - 25.2)	(0.0 - 59.2)	(4.1 - 8.9)
July	11.3	5.4	7.4	21.1	8.4
	(9.0 - 13.5)	(2.5 - 8.3)	(0.6 - 14.2)	(11.4 - 30.9)	(7.4 - 9.4)
August	7.0	8.3	5.5	12.6	7.2
	(5.6 - 8.3)	(6.3 - 10.3)	(0.0 - 12.0)	(3.0 - 22.1)	(6.2 - 8.2)
September	10.8	6.8	3.1	0.0	7.3
	(8.9 - 12.8)	(4.6 - 8.9)	(0.0 - 7.8)	(0.0 - 6.6)	(5.8 - 8.8)
October	7.4	9.3	3.4	1.3	6.5
	(5.3 - 9.6)	(7.6 - 10.9)	(0.9 - 5.9)	(0.0 - 4.7)	(5.1 - 7.8)
November	6.9	5.3	2.9	2.8	4.8
	(4.8 - 9.0)	(3.9 - 6.8)	(1.2 - 4.7)	(0.9 - 4.7)	(3.7 - 5.9)
December	6.3	3.8	2.5	2.0	3.8
	(5.1 - 7.6)	(3.0 - 4.6)	(1.5 - 3.6)	(0.9 - 3.2)	(3.2 - 4.4)
January	11.2	9.0	6.9	1.9	7.6
	(10.0 - 12.3)	(8.1 - 9.9)	(5.3 - 8.6)	(0.0 - 3.9)	(7.0 - 8.2)
February	8.9	12.2	20.0	2.4	9.4
	(5.4 - 12.5)	(8.3 - 16.2)	(12.4 - 27.6)	(0.0 - 9.5)	(7.8 - 11.0)
Average	8.0	7.6	5.9	5.8	6.8
	(6.0 - 10.4)	(4.9 - 10.4)	(2.4 - 12.2)	(1.8 - 16.1)	(5.6 - 8.1)

Note: the numbers in the table (k) can be interpreted as the number of infected out of which one case is detected. The interpretation can be as follows: Within a given month and stratum - one case out of every k cases was detected by the HSB. It is calculated as the total estimated cases using CoVIDA data divided by the total number of cases detected by the HSB the same period. Analytical Confidence Intervals in Parentheses.

Supplementary Table 4. Positivity Rate and Epidemiological week when occupation were able to resume work for the first time since the beginning of the pandemic (in week 12)

Occupation Group	Occupations Included	Positivity (%)	95% CI	Observations	Epidemiological Week* Allowed to Work
Administrative and Support	Secretary Staff (n= 3,105); Call Center Employees (n= 267); Insurance And Social Security Agent (n= 47); Travel Agency Employees (n= 35); Real Estate Agent (n= 24)	3.08	(2.51,3.65)	3,478	August 31st, 2020[1]
Architects and Engineers	Engineer (n= 1,678); Web Designer (n= 340); Computer Services Staff (n= 254); Electrician (n= 122); Mechanical Engineering Staff (n= 43)	2.05	(1.37,2.73)	2,437	August 31st, 2020[2]
Arts Entertainment and Recreation	Journalists And Writers (n= 667); Cultural Activities Staff (n= 613); Architects (n= 362); Artists (n= 196)	2.29	(1.50,3.07)	1,838	*Always*[3]
Construction	Construction Workers (n= 728); Seamstress And Related (n= 87); Carpenters And Related (n= 76); Shoemaker (n= 3)	4.03	(2.90,5.15)	894	April 20th, 2020[4]
Delivery Workers	Delivery Workers (n= 2,101)	3.00	(2.26,3.73)	2,101	*Always*
Educational Services	Student (n= 3,213); College Professor (n= 595); Secondary Teacher (n= 74); Preschool Teacher (n= 19); Primary Teacher (n= 11)	2.61	(2.07,3.15)	3,912	August 31st, 2020
Finance, Management, and Insurance	Personal Financial Services (n= 2,484); Directors And Managers Of Companies (n= 793)	3.08	(2.49,3.67)	3,277	*Always*[5]
Health Care and Social Assistance	Nurse (n= 2,271); Doctor (n= 1,936); Physiotherapist (n= 404); Dentists (n= 373); Medical Assistants (n= 236); Nutritionist (n= 61); Optometrist (n= 24); Hospital Admissions Staff (n= 14)	1.99	(1.53,2.45)	5,319	*Always*[6]
Lawyers	Lawyers (n= 979)	1.94	(0.87,3.02)	979	August 31st, 2020
Military, Police, and Firefighters	Police (n= 646); Military (n= 290); Firemen (n= 32); Air Force Officers (n= 6)	2.98	(1.90,4.06)	974	*Always*
Nannies, Maids, and Housekeeping Cleaners	Personal Grooming (n= 829); Trash Collectors (n= 107); Babysitter (n= 46); Car Washers (n= 4)	3.14	(2.07,4.22)	986	*Always*[7]
Professional, Scientific and Technical Services	Veterinarian (n= 507); Psychologists (n= 311); Biologist And Related (n= 253); Economists (n= 170); Sociologist, Anthropologist (n= 44); Chemical (n= 21); Geologist (n= 13); Political Scientist And Related (n= 9)	1.51	(0.58,2.43)	1,328	*Always*[8]
Retail Trade, Accommodation, and Food Services	Street Vendor (n= 1,406); Chefs (n= 205); Pharmacist (n= 199); Hairdressers And Related (n= 117); Waiter (n= 86); Baker And Related (n= 33); Shop Seller (n= 29)	6.17	(5.43,6.91)	2,075	August 31st, 2020[9]
Retired	Retired (n= 1,043)	2.30	(1.26,3.34)	1,043	August 31st, 2020
Security Guards	Security Guards (n= 1,211)	5.12	(4.15,6.09)	1,211	*Always*
Homemaker	Homemaker (n= 1,332)	4.35	(3.43,5.28)	1,332	August 31st, 2020
Taxi Drivers and Transportation	Taxi Drivers (n= 3,312); Personal Transportation (n= 423)	4.28	(3.73,4.83)	3,735	*Always*
Unemployed	Unemployed (n= 951)	4.00	(2.90,5.09)	951	August 31st, 2020
Not Classified	-	2.65	(2.14,3.17)	4,294	-

* Refers to the first day of the week in which the occupation with the most observations included in the occupation group was allowed to first work outside home since the beginning of the pandemic. 1. Call Centers were always open; 2. Electricians allowed to work outside home beginning on July 13th; 3. All but Journalists and Writers, allowed to work outside home beginning on August 31st; 4. All but Construction Workers, allowed to work outside home beginning on August 31st,2020; 5. CEOs were allowed to work outside home beginning on August 31st; 6. Dentists were allowed to work outside home beginning on August 31st; 7. Car Washers were allowed to work outside home beginning on August 31st; 8. All but Veterinarians and Psychologist, were allowed to work outside home beginning on August 31st,2020; 9. Hairdressers allowed to work beginning on July 13th.

Supplementary Table 5. Case Mortality Rate

Month	Avg. Daily Deaths	Avg. Daily Cases HSB	Avg. Daily Cases CoVIDA	Death Rate HSB	Death Rate CoVIDA
June	39.4	1,290	7,114	3.05	0.55
July	104	3,261	27,390	3.19	0.37
August	60	2,797	19,716	2.15	0.30
September	29.4	1,679	12,015	1.75	0.24
October	30	1,753	11,353	1.71	0.26
November	31	2,038	9,828	1.52	0.32
December	77.2	4,090	15,223	1.89	0.51
January	114.7	6707	28,416	1.71	0.23
Aggregated	54.3	2,675	14,934	2.03	0.36

Note: Data on deaths comes from HSB. The estimated cases using CoVIDA data were calculated using a monthly weighted average and assuming a 17 day positivity window. Weights were calculated based on workers' occupation. Population of workers category was obtained from a review of official records in several sources

Supplementary Table 6. Population by Locality

Locality	CoVIDA Data Population	CoVIDA Data %*	HSB Data Population	HSB Data %*	Official Population	Mean Stratum
Antonio Nariño	427	0.01	8,727	0.10	108,976	2.9
Barrios Unidos	1,007	0.01	13,101	0.16	276,453	3.4
Bosa	1,635	0.02	47,042	0.56	799,660	1.9
Chapinero	1,691	0.02	15,614	0.19	125,294	4.2
Ciudad Bolivar	1,229	0.01	38,909	0.46	776,351	1.4
Engativa	4,157	0.05	69,649	0.83	892,169	2.7
Fontibon	1,899	0.02	31,387	0.37	444,951	3.1
Kennedy	3,691	0.04	81,268	0.97	1,273,390	2.4
La Candelaria	165	0.00	2,937	0.04	21,830	2.4
Martires	346	0.00	8,236	0.10	92,234	2.9
Puente Aranda	1,339	0.02	25,624	0.31	211,802	3.0
Rafael Uribe Uribe	1,128	0.01	29,841	0.36	341,886	2.3
San Cristobal	1,198	0.01	30,055	0.36	387,560	2.1
Santa Fe	547	0.01	10,758	0.13	91,111	2.2
Suba	7,690	0.09	91,344	1.09	1,381,597	2.8
Teusaquillo	1,552	0.02	13,990	0.17	139,369	3.9
Tunjuelito	550	0.01	15,278	0.18	183,067	2.3
Usaquen	5,751	0.07	43,175	0.52	476,931	3.8
Usme	622	0.01	22,536	0.27	348,332	1.5

Note: Sumapaz was excluded from all estimations; *Percentage of official population taken from the Colombian National Statistical System (DANE in Spanish)

Supplementary Table 7. Population by Stratum

Stratum	CoVIDA Data Population	CoVIDA Data % *	HSB Data Population	HSB Data %*	Official Population
1 & 2	10,986	0.27	356,899	8.78	4,063,470
3	16,568	0.58	220,324	7.71	2,857,861
4	8,646	1.14	46,431	6.13	757,923
5 & 6	4,699	1.29	23,686	6.48	365,459

Note: *Percentage of official population taken from the Colombian National Statistical System (DANE in Spanish)

Supplementary Table 8. Positivity Rate by Age

Age	Positivity (%)	95% CI	Observations
18-29	3.09	(2.82,3.36)	12,022
30-39	2.89	(2.50,3.28)	9,940
40-49	3.15	(2.69,3.61)	7,148
50-59	3.01	(2.53,3.50)	5,475
60-70	3.20	(2.64,3.77)	2,423
70plus	3.38	(2.53,4.22)	713
No Info	3.18	(2.61,3.75)	4,440

Note: Table shows positivity rate, 95% confidence intervals and number of tests by age.The sample excludes individuals with symptoms or known contacts with an infected person.

Supplementary Table 9. Positivity Rate by Gender

Gender	Positivity (%)	95% CI	Observations
Female	3.07	(2.84,3.30)	20,068
Male	3.14	(2.89,3.39)	19,805
No Info	2.76	(2.09,3.43)	2,288

Note: Table shows positivity rate, 95% confidence intervals and number of tests by Gender.The sample excludes individuals with symptoms or known contacts with an infected person.

33 # Supplementary Note 4

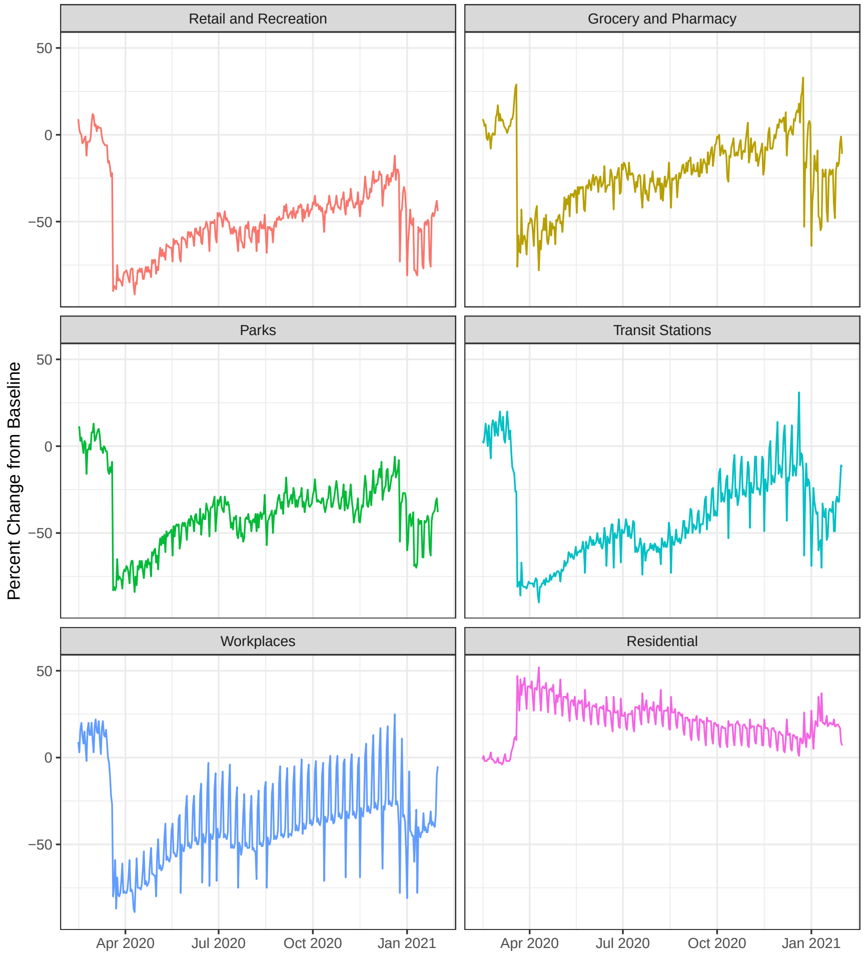

Supplementary Figure 6. **Mobility Changes by Location**. Data comes from Google's COVID-19 Community Mobility. Report Baseline is the median value, for the corresponding day of the week, during the 5- week period Jan 3–Feb 6, 2020

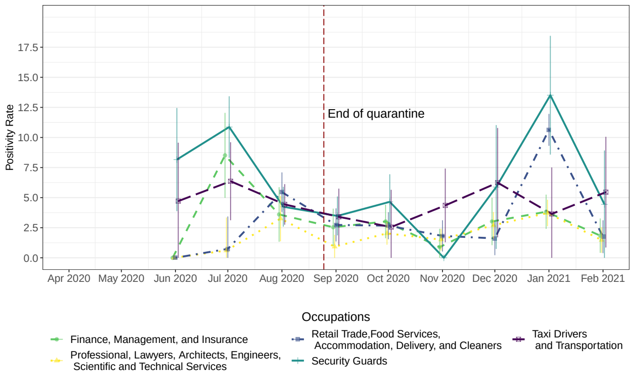

(a) Positivity by Occupation (CoVIDA data)

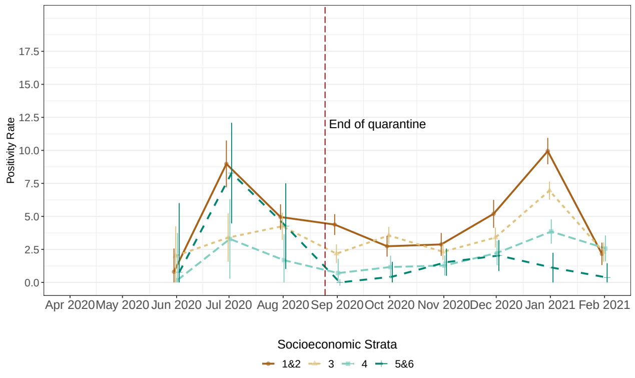

(b) Positivity by socio-economic Stratum (CoVIDA data)

Supplementary Figure 7. **Monthly Dynamics**. The Figure complements Figure 7 by adding 95% confidence intervals to the point estimations. Dots represent COVID-19 estimated monthly positivy rates (panels (a) and (b)) from CoVIDA data (n=42,164). Whiskers denote 95% confidence intervals. Weights are based on workers' occupation to be representative of Bogotá's population. Supplementary Tables 4 and 7 show a detailed decomposition by worker's occupation and stratum populations respectively.)

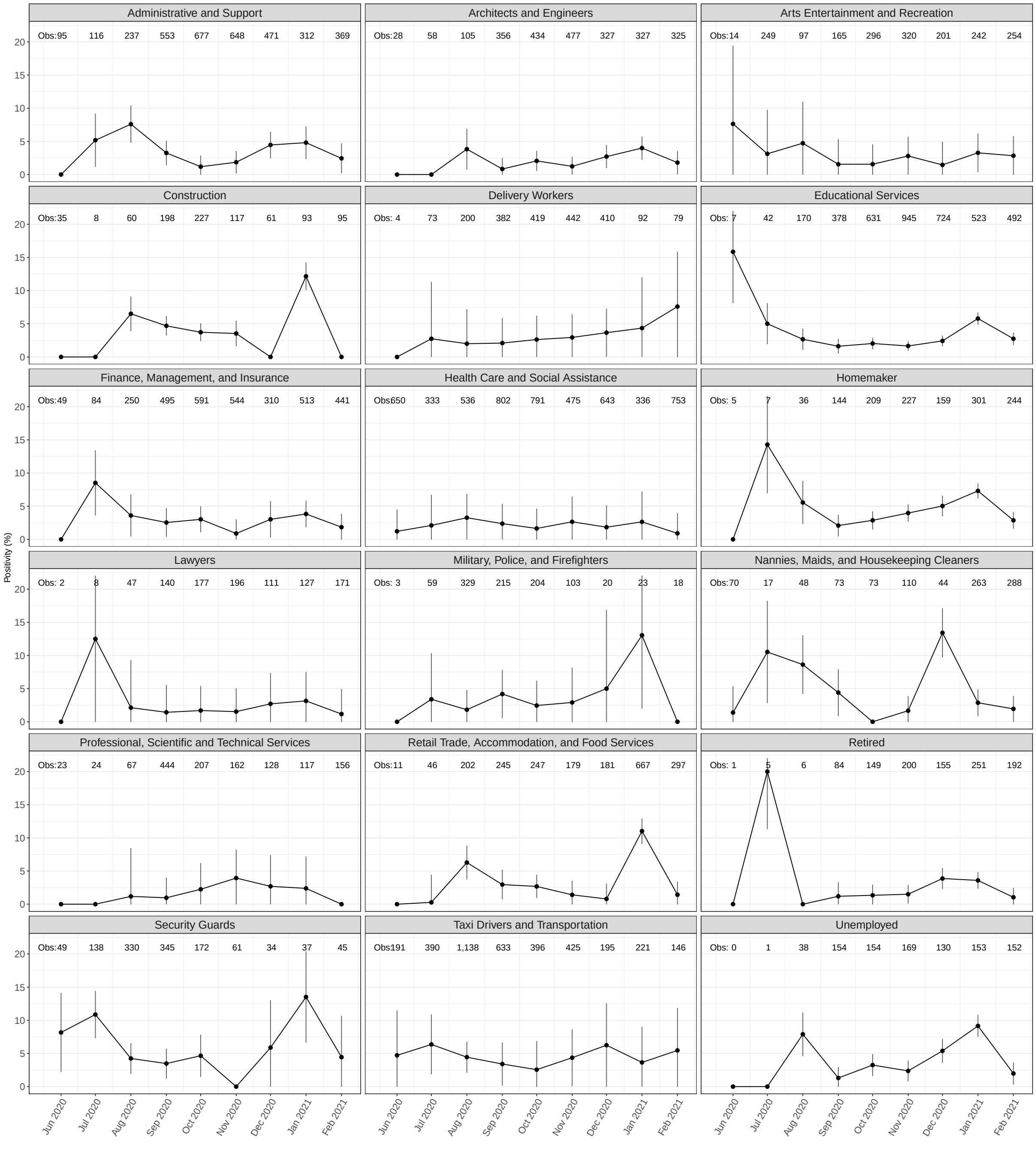

Supplementary Figure 8. **Monthly Dynamics by Occupation**. The Figure breaks up the occupational groups shown in Figure 3a. Dots represent estimated COVID-19 monthly positivity rates for each occupations described in Table 4. Whiskers denote 95% confidence intervals. The vertical dashed line marks the end of quarantine on August 25, 2020.

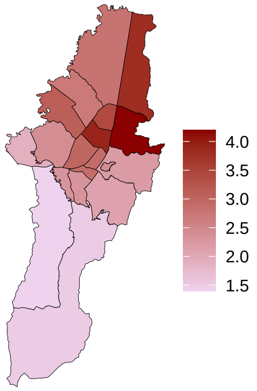

(a) Average Socio-economic Stratum

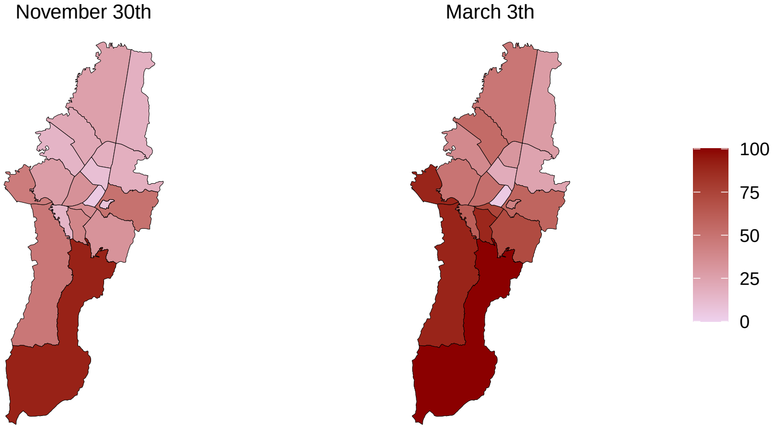

(b) CoVIDA data Accumulated Cases

Supplementary Figure 9. **Average Socio-economic Stratum and Dynamics by Locality** . This figure shows the accumulated COVID-19 cases in every locality of Bogotá using CoVIDA data. It was calculated using a weighted average positivity for the whole period. Weights were based on worker's occupation. Mean stratum was calculated based on official data from Bogotá's mayor office, it shows the average socio economic stratum by locality using the population of each stratum living in the locality. The base layer is public and was obtained from Infraestructura de Datos Espaciales of Bogotá (IDECA), available at https://mapas.bogota.gov.co/.

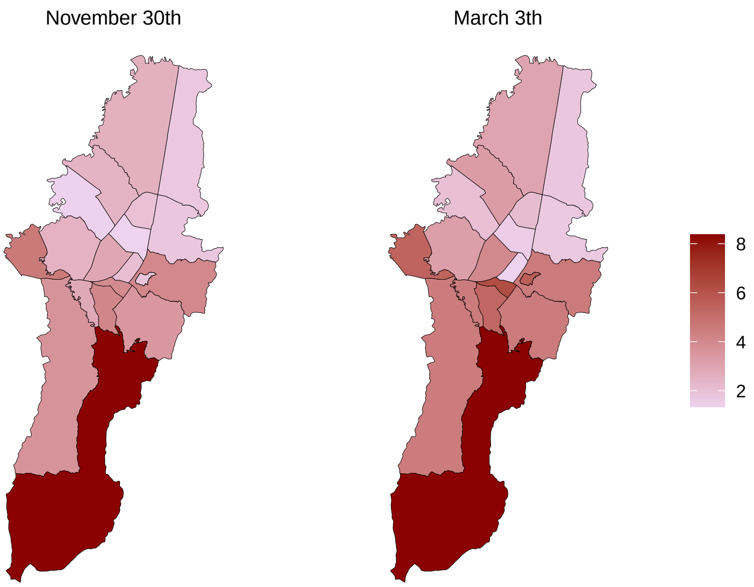

Supplementary Figure 10. **Raw Positivity by Locality**. This figure shows the unweighted raw COVID-19 positivity cases in every locality of Bogotá using CoVIDA data. The base layer is public and was obtained from Infraestructura de Datos Espaciales of Bogotá (IDECA), available at https://mapas.bogota.gov.co/.

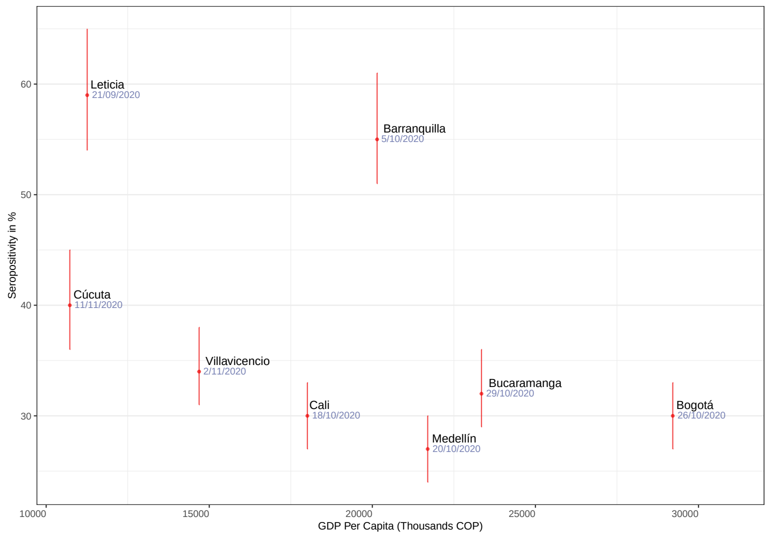

(a) Seropositivity

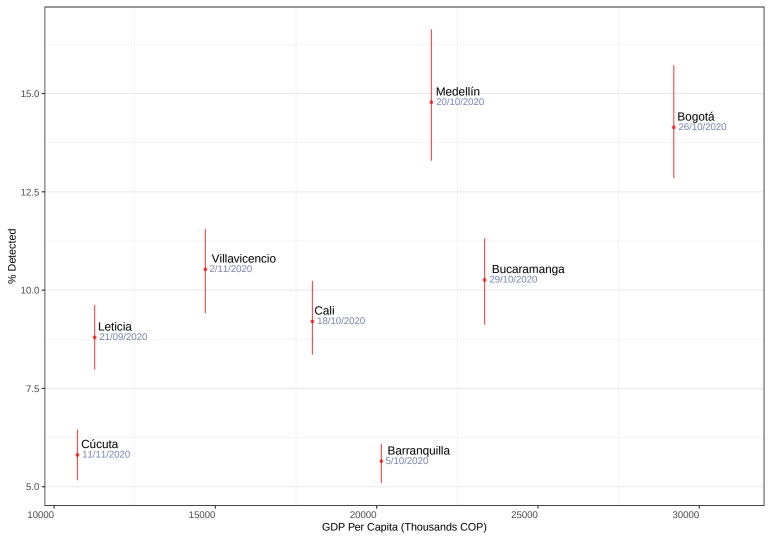

(b) Detection Rate

Supplementary Figure 11. **COVID-19 Seropositivity and Detection Rate across Colombia**. This figure shows the detection rate in other cities of Colombia using official data from the National Health Institute of Colombia (NHI). Calculations ere performed using the adjusted seropositivity estimates from NHI. For the case of Bogotá we adjusted the total number of cases to those reported to us by the Health Secretary of Bogotá (HSB). Whiskers denote 95% confidence intervals. . Total number of observations for seropositivity calculation varies by city and comes directly from NHI; for Cúcuta n=1,453, Leticia n=1,417, Villavicencio n=1,457, Cali n=1,979, Barranquilla n=1,487, Medellín n=1,832, Bucaramanga n=1,429, Bogotá n=4,597.

Supplementary References

[1] Instituto Nacional de Salud. Estudio nacional de seroprevalencia. *Estudio Nacional de Seroprevalencia* (2020). URL https://www.ins.gov.co/estudio-nacional-de-seroprevalencia/reporte.html#curso.

[2] Wallinga, J. & Lipsitch, M. How generation intervals shape the relationship between growth rates and reproductive numbers. *Proceedings of the Royal Society B: Biological Sciences* **274**, 599–604 (2007).

[3] Laajaj, R. *et al.* Understanding inequalities in sars-cov-2 infections across socioeconomic groups (2020). Mimeo.

[4] He, X. *et al.* Temporal dynamics in viral shedding and transmissibility of covid-19. *Nature medicine* **26**, 672–675 (2020).

[5] McAloon, C. *et al.* Incubation period of covid-19: a rapid systematic review and meta-analysis of observational research. *BMJ Open* **10** (2020). URL https://bmjopen.bmj.com/content/10/8/e039652. https://bmjopen.bmj.com/content/10/8/e039652.full.pdf.

[6] Challen, R., Brooks-Pollock, E., Tsaneva-Atanasova, K. & Danon, L. Meta-analysis of the sars-cov-2 serial interval and the impact of parameter uncertainty on the covid-19 reproduction number. *medRxiv* (2020). URL https://www.medrxiv.org/content/early/2020/11/20/2020.11.17.20231548. https://www.medrxiv.org/content/early/2020/11/20/2020.11.17.20231548.full.pdf.

[7] Hilton, J. & Keeling, M. J. Estimation of country-level basic reproductive ratios for novel coronavirus (sars-cov-2/covid-19) using synthetic contact matrices. *PLoS computational biology* **16**, e1008031 (2020).
