# Supplementary material for: COVID-19 spread, detection, and dynamics in Bogota, Colombia
Source: Nat Commun. 2021 Aug 5;12:4726. doi: 10.1038/s41467-021-25038-z (PMC8342514; doi:10.1038/s41467-021-25038-z)
Supplement: Supplementary file 3 — Reporting Summary [file 41467_2021_25038_MOESM3_ESM.pdf]

nature portfolio

Corresponding author(s): Rachid Laajaj

Last updated by author(s): 20021-06-09

# Reporting Summary

Nature Portfolio wishes to improve the reproducibility of the work that we publish. This form provides structure for consistency and transparency in reporting. For further information on Nature Portfolio policies, see our Editorial Policies and the Editorial Policy Checklist.

## Statistics

For all statistical analyses, confirm that the following items are present in the figure legend, table legend, main text, or Methods section.

| n/a | Confirmed | |
|---|---|---|
| ☐ | ☒ | The exact sample size (*n*) for each experimental group/condition, given as a discrete number and unit of measurement |
| ☐ | ☒ | A statement on whether measurements were taken from distinct samples or whether the same sample was measured repeatedly |
| ☐ | ☒ | The statistical test(s) used AND whether they are one- or two-sided<br>*Only common tests should be described solely by name; describe more complex techniques in the Methods section.* |
| ☐ | ☒ | A description of all covariates tested |
| ☐ | ☒ | A description of any assumptions or corrections, such as tests of normality and adjustment for multiple comparisons |
| ☐ | ☒ | A full description of the statistical parameters including central tendency (e.g. means) or other basic estimates (e.g. regression coefficient) AND variation (e.g. standard deviation) or associated estimates of uncertainty (e.g. confidence intervals) |
| ☐ | ☒ | For null hypothesis testing, the test statistic (e.g. $F$, $t$, $r$) with confidence intervals, effect sizes, degrees of freedom and $P$ value noted<br>*Give P values as exact values whenever suitable.* |
| ☒ | ☐ | For Bayesian analysis, information on the choice of priors and Markov chain Monte Carlo settings |
| ☒ | ☐ | For hierarchical and complex designs, identification of the appropriate level for tests and full reporting of outcomes |
| ☒ | ☐ | Estimates of effect sizes (e.g. Cohen's *d*, Pearson's *r*), indicating how they were calculated |

*Our web collection on statistics for biologists contains articles on many of the points above.*

## Software and code

Policy information about availability of computer code

| | |
|---|---|
| Data collection | We used Salesforce program for data collection: a personalized version of Salesforce Health Cloud and Salesforce Communities (version 2020). All survey instruments are avaliable at OPENICPSR under accession code 142121, available at: https://www.openicpsr.org/openicpsr/project/142121/version/V1/view |
| Data analysis | The data analysis was conducted using Stata-16 version 16.1 and R version 4.0.2 (2020-06-22) |

For manuscripts utilizing custom algorithms or software that are central to the research but not yet described in published literature, software must be made available to editors and reviewers. We strongly encourage code deposition in a community repository (e.g. GitHub). See the Nature Portfolio guidelines for submitting code & software for further information.

## Data

Policy information about availability of data

All manuscripts must include a data availability statement. This statement should provide the following information, where applicable:

- Accession codes, unique identifiers, or web links for publicly available datasets
- A description of any restrictions on data availability
- For clinical datasets or third party data, please ensure that the statement adheres to our policy

The data used in this manuscript comes from the CoVIDA project. led by the University of Los Andes and from administrative data of the Health Secretary of Bogota. The processed data sets are available at OPENICPSR under accession code 142121 all the date is available at: https://www.openicpsr.org/openicpsr/project/142121/version/V1/view

The codes that support all the findings of this study are available at:
https://github.com/ignaciomsarmiento/spread_detection_and_dynamics_CoVIDA

# Field-specific reporting

Please select the one below that is the best fit for your research. If you are not sure, read the appropriate sections before making your selection.

☒ Life sciences ☐ Behavioural & social sciences ☐ Ecological, evolutionary & environmental sciences

For a reference copy of the document with all sections, see nature.com/documents/nr-reporting-summary-flat.pdf

# Life sciences study design

All studies must disclose on these points even when the disclosure is negative.

| | |
|---|---|
| Sample size | The data include 59,770 PCR tests in the full sample from CoVIDA primary data. An objective of approximately 60,000 tests was decided at the onset of the project ensuring enough power to draw conclusions even when the sample is split in various groups, and complying with the budget restriction of the project. |
| Data exclusions | In the main results, we excluded data from cases that were symptomatic and with a known contact with a positive person. This exclusion was pre-established to avoid self-selection from peopole who believe that they may be positive.<br>Additionaly, we excluded a military batalion in which we had an outbreak of almost 50% positivity. This exclusion was not pre-established, but this positivity rate drives up the monthly average and is not representative of the full sample of military workers. Results without data exclusion are shown in the Supplementary Information. |
| Replication | All the results are replicable. Our attempt to replicate the results, with the same data and code, from a different person in a different computer was successful. |
| Randomization | We applied a random selection of participants to be invited when lists of potential participants that were provided through agreeements exceeded our capacity to test them. In the case of health workers, the random selection was stratified by high exposure and low exposure in order to keep a balance between these two groups. Other lists were not stratified (no covariates were used to determine participation to the study). This randomization for participation in the study was done with Stata software and all the codes were preserved. Our testing and data collection was observational, hence there was no randomization into an experimental and control group. |
| Blinding | Blinding is not relevant to our study because it has no experimental and control group. |

# Reporting for specific materials, systems and methods

We require information from authors about some types of materials, experimental systems and methods used in many studies. Here, indicate whether each material, system or method listed is relevant to your study. If you are not sure if a list item applies to your research, read the appropriate section before selecting a response.

## Materials & experimental systems

| n/a | Involved in the study | |
|---|---|---|
| ☒ | ☐ | Antibodies |
| ☒ | ☐ | Eukaryotic cell lines |
| ☒ | ☐ | Palaeontology and archaeology |
| ☒ | ☐ | Animals and other organisms |
| ☐ | ☒ | Human research participants |
| ☒ | ☐ | Clinical data |
| ☒ | ☐ | Dual use research of concern |

## Methods

| n/a | Involved in the study | |
|---|---|---|
| ☒ | ☐ | ChIP-seq |
| ☒ | ☐ | Flow cytometry |
| ☒ | ☐ | MRI-based neuroimaging |

## Human research participants

Policy information about studies involving human research participants

| | |
|---|---|
| Population characteristics | Participants are residents of Bogota that are 14 years and above. They were contacted as part of our support of the community epidemiological surveillance done together with the Health Secretary of Bogota. Some occupations were prioritized in the invitation messages, but we did not restrict participation to any gender, occupation or any other covariate. |
| Recruitment | Participants were randomly selected from lists (mainly organized by occupation) to be invited through phone callse, and others came from public invitations in media communications.<br>This generates an over or under-representation of some occupations and potential risk of selection bias from those that are concerned of being positive. To account for this, we re-weight by occupation and eliminate cases with symptoms or known contact (reducing risk of self-selection because one may believe that he/she may be infected). |

| | |
|---|---|
| Ethics oversight | Ethics approval was obtained from the ethics committee of Universidad de los Andes (Number 1181, 2020) |

Note that full information on the approval of the study protocol must also be provided in the manuscript.
